# Association between Nutritional Status and Mortality after Aortic Valve Replacement Procedure in Elderly with Severe Aortic Stenosis

**DOI:** 10.3390/nu11020446

**Published:** 2019-02-20

**Authors:** Edyta Wernio, Sylwia Małgorzewicz, Jolanta Anna Dardzińska, Dariusz Jagielak, Jan Rogowski, Agnieszka Gruszecka, Andrzej Klapkowski, Peter Bramlage

**Affiliations:** 1 Department of Clinical Nutrition, Medical University of Gdańsk, 80-211 Gdańsk, Poland; sylwia.malgorzewicz@gumed.edu.pl (S.M.); annadar@gumed.edu.pl (J.A.D.); 2 Department of Cardiac and Vascular Surgery, Medical University of Gdańsk, 80-211 Gdańsk, Poland; dariusz.jagielak@gumed.edu.pl (D.J.); jan.rogowski@gumed.edu.pl (J.R.); a.klapkowski@gumed.edu.pl (A.K.); 3 Department of Radiology Informatics and Statistics, Medical University of Gdansk, 80-211 Gdansk, Poland; agnieszka.gruszecka@gumed.edu.pl; 4 Institute for Pharmacology and Preventive Medicine, 49661 Cloppenburg, Germany; peter.bramlage@ippmed.de

**Keywords:** nutritional status, mortality, severe aortic stenosis

## Abstract

**Background:** There is still a lack of data on the nutritional status of older people with aortic stenosis (AS) and the effect of poor nutrition on the occurrence of complications and mortality after an aortic valve replacement (AVR) procedure. The aim of this study was to assess the impact of selected nutritional status parameters in elderly patients with severe AS on the occurrence of postoperative complications and one-year mortality after the AVR procedure. **Methods:** 101 elderly patients with AS aged 74.6 ± 5.2 years who qualified for surgical treatment (aortic valve area [AVA] 0.73 ± 0.2 cm^2^) were enrolled in the study. A nutritional status assessment was performed before AVR surgery, and the frequency of postoperative complications occurring within 30 days of surgery was assessed. The one-year mortality rate was also captured. **Results:** Adverse events (both major and minor) up to 30 days occurred in 49.5% (*n* = 50) of the study population. Low Mini Nutritional Assessment (f-MNA) and Subjective Global Assessment (7-SGA) scores and low concentrations of total cholesterol, LDL-cholesterol, and prealbumin were associated with a higher risk of postoperative complications. The risk of complications increased 1.22 times (95% CI; 1.030–1.453; *p* = 0.019) with an impaired nutritional status. The annual mortality rate in the study group was 7.9%. Unintentional weight loss of >2.8% in the six months preceding surgery proved useful for predicting death within the first year after AVR surgery. **Conclusions:** The results indicate that poor nutritional status is an important factor affecting the adverse outcomes in elderly patients with severe aortic valve stenosis undergoing an AVR procedure.

## 1. Introduction

We are obliged to elucidate that the presented article is a continuation of previous research published in this journal [1]. Due to the consideration of numerous data, it was decided to present the results in a separate paper.

Physiological changes that occur with the aging process predispose elderly people to a deterioration in their nutritional status, as well as to the development of geriatric syndromes. In most cases, the risk of occurrence of these nutritional abnormalities is compounded by associated diseases and disease-related pharmacotherapy [2,3,4]. Malnutrition, sarcopenia, frailty syndrome, and anorexia are more likely to be diagnosed in elderly individuals with multiple chronic diseases, as well as in those who are hospitalized or are staying in nursing homes. It is well documented that malnutrition can aggravate the prognosis of older patients and contribute to increasing healthcare costs [5]. 

Aortic stenosis (AS) is the most common valve lesion in the older population. In the face of the rapid growth in the number of elderly people in Europe, it is likely that the number of new diagnoses of AS will also increase [6,7]. Currently, the only effective treatment for this progressive valve disease is aortic valve replacement (AVR), which is associated with complications and postoperative mortality. Aside from the well-known risk factors, such as advanced age, multiple comorbidities, preoperative state, or cardiac-related factors, increasing attention is now being paid to the patient’s nutritional status [7,8]. Despite many assumptions, there is still a lack of data on the nutritional status of elderly people prior to AVR and the effect of poor nutrition on the occurrence of complications and mortality after the procedure. 

The aim of this study was to assess the impact of selected nutritional status parameters on the occurrence of postoperative complications and one-year mortality after the AVR procedure. 

## 2. Materials and Methods

The study enrolled 101 elderly patients with severe AS. Patients were eligible for inclusion in the study if they had been diagnosed with severe AS based on echocardiogram results (aortic valve area [AVA] <1 cm^2^ or 0.6 cm^2^/m^2^ body surface area, mean gradient >40 mm Hg or peak aortic velocity >4.0 m/s); were aged 65 years or older; had a lack of unstable, severe, concomitant disease (such as pulmonary disease, hepatic insufficiency, or kidney insufficiency (Kidney Disease Outcomes Quality Initiative (K/DOQI) stage >3, meaning eGFR <30 mL/min/1.73 m^2^); and provided written consent for participation in the study. Patients were excluded from the study if they were less than 65 years; had only mild or moderate AS; had disabled cognitive function; an impaired cardioverter defibrillator; or had a severe disease (including kidney failure, severe chronic obstructive lung disease, liver cirrhosis, or a positive history of cancer). 

All subjects were routinely admitted for the AVR, with or without other accompanied interventions. The procedure was performed in the Department of Cardiac and Vascular Surgery, University Clinical Center in Gdansk, Poland. The consent to conduct research was obtained from the University of Gdansk Ethics Committee.

### 2.1. Anthropometry

The mechanical column scale (Seca 711) was used to determine the body weight (kg), and height (cm) was measured using a telescopic stadiometer (Seca 220). The following body mass index (BMI) categories were adopted from the Committee on Diet and Health for elderly: low BMI (<24 kg/m^2^), normal BMI (24–29 kg/m^2^), and high BMI (>29 kg/m^2^) [9]. An analogue hand-held dynamometer (Baseline 12-0240, Burr Ridge, IL, USA) was used to assess the handgrip strength (HGS). The HGS reference value was adopted in accordance with The European Working Group on sarcopenia in older people [10]. Phase angle (50 kHz) was evaluated by the bioelectrical impedance analysis (BIA) method (Maltron BioScan 920-2, Rayleigh, UK). The percentage of unintentional weight loss within 6 months was also calculated.

### 2.2. Nutritional Status

Evaluation of the nutritional status was carried out using the full version of the Mini Nutritional Assessment (f-MNA) and the 7-point Subjective Global Assessment (7-SGA). According to the f-MNA scale, 24–30 points indicates that a patient has a good nutritional status, 17–23.5 points indicates that a patient is at risk of malnutrition, and <17 points suggests the patient is malnourished [11]. According to the 7-SGA scale, 1–3 points classifies the patient as malnourished, 4–5 points indicates the patient is moderately malnourished, and 6 or 7 points indicates the patient is well-nourished [12]. The risk of deterioration of the nutritional status associated with a decrease in appetite has been investigated using the Simplified Nutritional Assessment Questionnaire (SNAQ), with ≤14 points indicating decreased appetite and a risk of weight loss within the next six months [13].

### 2.3. Comorbidity Index

Patient comorbidity data was captured using the Charlson Comorbidity Index (CCI). The age-adjusted CCI was calculated according to the reference method. CCI takes into consideration 19 diseases with a suitable weight. Additional points are added for each decade after 40 years of age (1 point for ages 41–50, 2 points for ages 51–60, 3 points for ages 61–70, and 4 points for ≥71 years of age) [14].

### 2.4. Biochemistry

On the day preceding the intervention, while the patient was hospitalized, a blood sample was collected from all subjects in a fasting state for biochemical analysis. Biochemical parameters were estimated by routine laboratory procedures before surgery and included concentration of albumin (g/L), prealbumin (mg/dL), C-reactive protein (CRP, mg/dL), red blood cells (×10^9^/L), hemoglobin (g/dL), white blood cells (×10^9^/L), complete blood count (in 1 mm^3^), LDL-cholesterol (mg/dL), total cholesterol (mg/dL), and HDL-cholesterol (mg/dL).

### 2.5. Postoperative Complications/Adverse Events and Mortality

All complications (major and minor) that occurred up to 30 days after the AVR were recorded. Data on one-year mortality after the AVR were obtained from the Polish National Health Fund.

### 2.6. Statistics

The statistical analysis was performed with Statistica 13.1 for Windows. Distribution of variables was assessed with the Shapiro–Wilk test. Data are presented as the mean ± standard deviation (SD) or median and range. A *p*-value of <0.05 was considered to be statistically significant. Depending on the data distribution, a Pearson and/or Spearman test analysis of correlations was conducted. The receiver operating characteristic (ROC) curves were used to assess the predictive value of selected quantitative variables on the occurrence of complications and one-year mortality. Univariate logistic regression analysis was performed for selected variables. Depending on the distribution of variables, the T-test or Mann–Whitney U test was used in a comparative analysis of the two groups.

## 3. Results

Data were analyzed for 101 patients who were qualified for and underwent surgical procedures for severe AS. Briefly, the mean age of study participants was 74.6 years. Patients had a mean AVA of 0.73 ± 0.20 cm^2^ and a mean EUROScore II of 1.98 (only one patient who qualified for transcatheter aortic valve implantation received 72 points). More than half of the patients received AVR alone, while the remaining patients underwent AVR with additional surgery, including coronary artery bypass grafting (CABG), mitral valve replacement (MRV), or a combination of both procedures. All patients suffered from a chronic disease and therefore were chronically treated. The median number of medications taken was five. Other patient demographics at baseline are presented in Table 1. In addition to the comorbidities included in Table 1, patients with AS experienced diseases noted in CCI, such as peptic ulcer disease (4% of subjects), mild liver disease (1% of subjects), rheumatoid arthritis (1% of subjects), and hemiplegia (1% of subjects).

As we reported previously [1], a significant number of patients before the AVR procedure was malnourished or at risk of malnutrition (44% of patients according to the f-MNA scale and 42% of patients according to the 7-SGA scale) [1]. Similarly, loss of appetite was diagnosed in 27% of older people before the surgical procedure. In addition, 36% of the study population had decreased HGS, 41% of patients had low albumin levels (<35 g/L), and 5% of patients had a decreased prealbumin concentration (<19.5 mg/dL). Other nutritional status data and biochemical parameters in patients at the beginning of the study are presented in Table 2.

### 3.1. The Postoperative Complications

Major and minor postoperative complications occurring up to 30 days after the AVR procedure occurred in 49.5% (*n* = 50) of study participants. Among the minor complications, the most frequent were paroxysmal atrial fibrillation (*n* = 15), other arrhythmias (*n* = 13), and delirium (*n* = 8), while less common complications included pericardial effusion (*n* = 3), pleural effusion (*n* = 2), and pneumothorax (*n* = 1). Serious postoperative complications were noted in 21 patients (21%) and included tamponade (*n* =5), bleeding (*n* = 4), chronic kidney disease (*n* = 3), respiratory failure (*n* = 3), pneumonia (*n* =1), low cardiac output syndrome (*n* = 1), third-degree atrioventricular block (*n* = 1), exacerbation of chronic obstructive pulmonary disease (*n* = 1), aortic dissection (*n* = 1), heart failure (*n* =1), and asystole (*n* = 1). Among those participants who developed severe complications, four patients (4%) died within 30 days after their AVR. 

**Figure 1:**
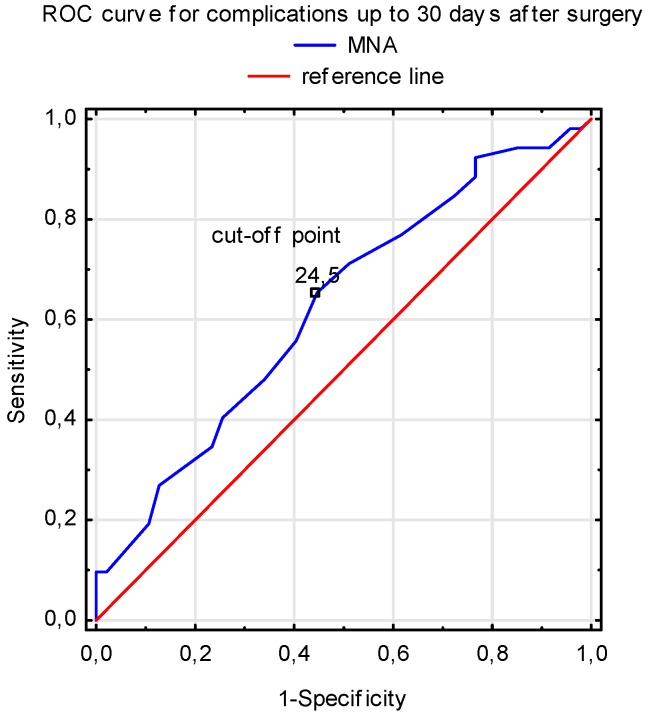
The predictive value of f-MNA results for surgical complications up to 30 days after AVR (sensitivity = 0.654; specificity = 0.553).

The ROC analysis revealed that nutritional status, as assessed by both the f-MNA and 7-SGA scales before surgery, possessed predictive values for complications up to 30 days after the AVR. Malnutrition (f-MNA and 7-SGA results) was associated with a higher risk of 30-day postsurgical complications (Table 3). The f-MNA scale cut-off for increased risk of complications was 24.5 points (Figure 1). Additionally, logistic regression analysis showed that the risk of postoperative complications increased 1.22 times (95% CI: 1.030–1.453; p = 0.019) with impaired nutritional status before surgery, as determined by the f-MNA scale.

**Figure 2:**
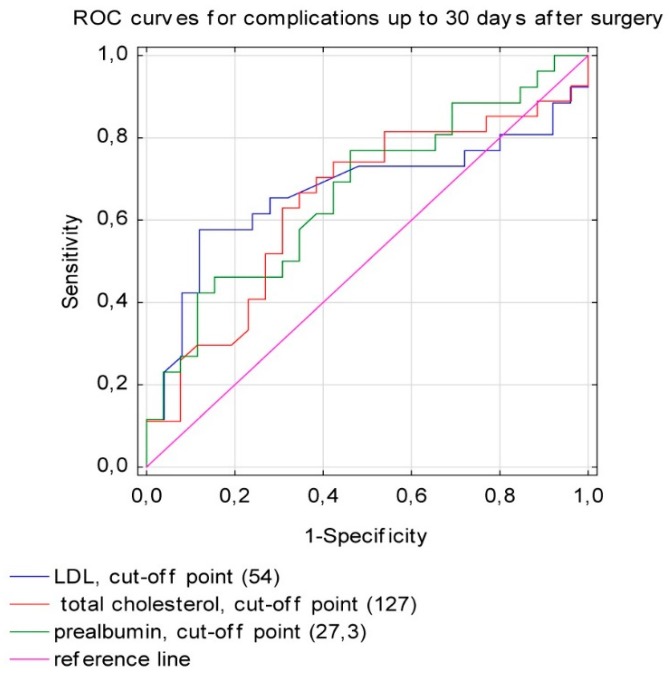
The predictive value of LDL-cholesterol, total cholesterol, and prealbumin for surgical complications up to 30 days after AVR (LDL cholesterol sensitivity = 0.577, specificity = 0.880; total cholesterol sensitivity = 0.630, specificity = 0.692; prealbumin sensitivity = 0.462, specificity = 0.846).

The high risk of postoperative complications was significantly associated with low concentrations of total cholesterol, LDL-cholesterol, and prealbumin before surgery (Figure 2 and Table 4). Percentage left ventricular ejection fraction (LVEF) was a predictive parameter for postoperative complications (area under the curve [AUC] 0.369; 95% CI: 0.252–0.485; *p* = 0.033). Other variables, including phase angle, HGS, or albumin level, did not show a prognostic value. 

Making a comparison of the baseline parameters of patients who developed postoperative complications (complicated patients) versus patients without postsurgical complications (uncomplicated patients) showed that patients who developed complications had a significantly worse nutritional status (f-MNA, 23.7 ± 2.7 vs. 25 ± 2.3, *p* = 0.033; 7-SGA, 5 (2–6) vs. 6 (3–6), *p* = 0.034) and significantly lower prealbumin (29.73 ± 8.23 vs. 34.4 ± 7.7, *p* = 0.038) and LDL-cholesterol concentrations (53.5 (3–127) vs. 71 (43–112), *p* = 0.039) than patients with no postsurgical complications (Table 5).

### 3.2. The Mortality Rate

**Figure 3:**
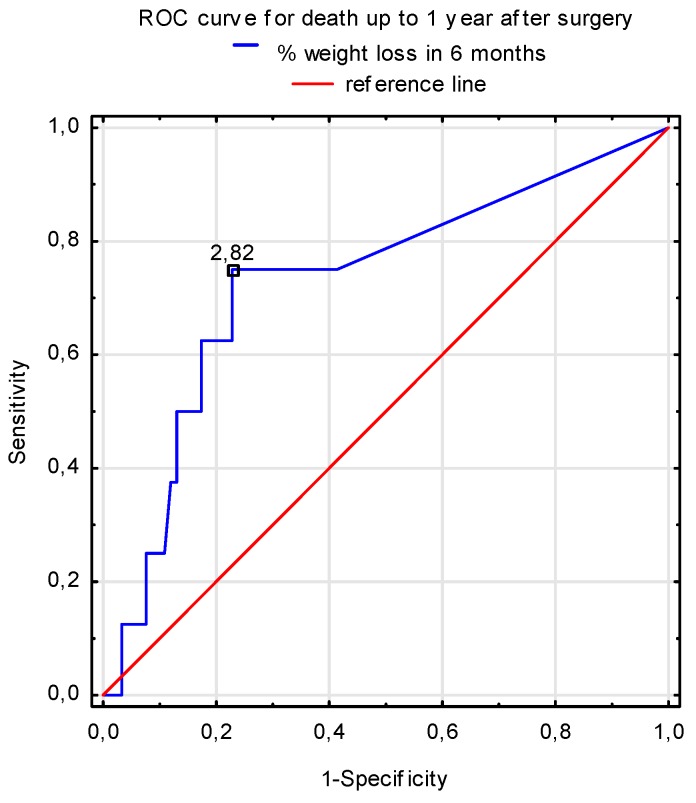
Percentage of unintentional weight loss within six months as a prognostic parameter of one-year mortality in elderly after AVR (sensitivity = 0.750, specificity = 0.772).

The one-year mortality rate in the study group was 7.9%, with the cause of death being sepsis (*n* = 3), sudden cardiac arrest (*n* = 2), multiorgan failure (*n* = 2), and chronic respiratory failure (*n* = 1). The ROC analysis showed that the percentage of unintentional weight loss prior to AVR surgery was an independent predictor of death within the first year of surgery. As the percentage of weight loss increased, the risk of death up to one year after surgery also increased. The proposed cut-off point for weight loss increasing the risk of death was a loss of 2.8% of total body weight in the preceding six months (Figure 3). Logistic regression analysis showed that the risk of one-year mortality after AVR surgery in elderly patients with severe AS increased 1.27 times (95% CI: 1.02–1.59; *p* = 0.0322) with an increased percentage of body weight loss. 

## 4. Discussion

The results of this study show that in elderly patients with severe AS, their nutritional status plays an important role in the outcome of AVR procedures. Determinants such as malnutrition/the risk of malnutrition decreasing levels of prealbumin, total cholesterol and LDL-cholesterol are important in predicting the occurrence of AVR complications. Furthermore, unintentional weight loss within the six months prior to surgery was significantly associated with an increased risk of one-year mortality after the AVR procedure. Our results indicate that it is necessary to assess the nutritional status of elderly patients with severe AS before they undergo surgery and that this should become a routine element of the preoperative procedure. 

Aortic valve stenosis is the most common valve lesion in older people [15], and it is well documented that this chronic, progressive disease with a prolonged inflammatory process may contribute to the patient’s reduced mobility, loss of muscle mass, and decreased appetite [16,17]. In our previous study, we showed that the deterioration of nutritional status is widespread in patients with symptomatic severe AS [1]. Besides a lower f-MNA and 7-SGA score, elderly patients typically had a worse appetite, lower muscle strength, and unintentional weight loss in comparison with healthy older people. Moreover, the presence of AS increased the risk of deterioration of their nutritional status between 3.6–8.5 times [1]. These findings are highly relevant, as many studies have shown that poor nutrition prior to cardiac surgery is associated with a higher risk of complications and a poorer prognosis after the procedure [18,19,20,21]. Additionally, an observational cohort study showed that the combination of a nutritional status assessment with the European System for Cardiac Operative Risk Evaluation (EuroSCORE) significantly improved the prediction of complications, in-hospital mortality, and 30-day mortality after cardiac surgery [18].

The majority of elderly patients included in this study were characterized by low perioperative risk, but major and minor adverse events occurred in 49.5% of subjects, and the signs of inappropriate nutrition were observed in almost 50% of subjects. Both the f-MNA and 7-SGA assessment tools possess a predictive value for complications [11,12]. In this study, we found a slightly higher prognostic value with the f-MNA scale and showed that the cut-off for increased operative risk was 24.5 points. These patients were still considered to be in a proper albeit borderline state of nutrition, and it is possible that perioperative stress could have affected the deterioration of their nutritional status. Eichler et al. [19] demonstrated that for each additional point in the MNA, patients after transcatheter aortic valve implantation (TAVI) had a reduced mortality risk of 17% within 12 months [19]. It seems that, especially in elderly, a multifactorial approach is needed for the preparation of patients for cardiac surgery, and this approach can also assist with predicting successful outcomes of the patient’s treatment plan [19].

Our results indicate also that preoperative, decreased serum concentrations of prealbumin, total cholesterol, and LDL-cholesterol were related to negative outcomes after AVR surgery. Prealbumin is a protein with a short half-life of two days in plasma which responds quickly to changes in protein-energy status and may reflect dietary intake [22,23,24]. Other studies have also noted that the lower preoperative concentration of prealbumin leads to adverse outcomes after cardiac surgery [25,26]. Interestingly, in our study, the cut-off point of prealbumin concentration for an increased complication risk was 27.3 mg/dL, while the normal levels of prealbumin are considered to be 19.5–35.8 mg/dL. 

It is well established that low plasma total cholesterol and LDL-cholesterol are sensitive markers of quantitative/qualitative nutritional deficiency in hospitalized patients [27,28,29]. Fröhlich et al. [28] demonstrated that independently of statin therapy, total cholesterol concentration of ≤3.6 mmol/L was associated with worse survival in patients with nonischaemic chronic heart failure [28]. In our study, elderly patients with severe AS had comorbidities, a worse nutritional status, and a lower LDL-cholesterol level at baseline. Moreover, an LDL-cholesterol level of ≤54 mg/dL and total cholesterol level of ≤127 mg/dL had a predictive value for the occurrence of complications.

Approximately 44% of patients with AS in our study experienced unintentional weight loss before the AVR procedure. The risk of one-year mortality after AVR was 27% higher in those elderly patients who reported an unintentional body weight loss. A seemingly insignificant reduction of 2.8% of total body weight within six months before AVR surgery proved to be a prognostic factor of mortality after the AVR procedure. Involuntary weight loss is regarded as an independent marker of poor outcomes in older people with chronic diseases. In a group of 6,933 patients with heart failure, a ≥5% reduction of body weight in six months increased the risk of mortality by approximately 50%, both from cardiovascular and other causes of death compared with those with stable weight [30].

The limitation of our study is that it included a relatively small number of patients. Nevertheless, we have proven that elderly patients often have an impaired nutritional status, and this is something that needs to be taken into consideration when preparing patients for AVR surgery. Furthermore, the physiological stress that accompanies surgery may aggravate the patient’s malnutrition, which increases the risk of an adverse outcome. For these reasons, nutritional support therapy (e.g., dietary counselling, oral supplementation) or even the introduction of an enhanced recovery after cardiac surgery (ERACS) protocol should be considered when treating this group of patients [31,32].

## 5. Conclusions

The results of this study indicate that the assessment of the patient’s nutritional status (including parameters such as total cholesterol, LDL-cholesterol, and prealbumin), as well as the use of the f-MNA and 7-SGA assessment tools prior to AVR surgery, may be a valuable prognostic marker of surgical complications. Moreover, unintentional weight loss in the six months preceding surgery appears to be useful in the prognosis of one-year mortality after AVR.

## Figures and Tables

**Table 1:** Characteristics of patients with severe aortic stenosis (AS) before surgery (*n* = 101).

Parameters (Mean ± SD or Median and Range)	Patients with AS (*n* = 101)
Age (years)	74.6 ± 5.2
Age-adjusted CCI	4 (2–9)
Length of hospital stay (days)	9.96 ± 5
Number of medications taken	5 (2–11)
Female/male (n)	48/53
AVA (cm^2^)	0.73 ± 0.2
Mean gradient (mm Hg)	47.4 ± 12.9
Peak aortic velocity (m/s)	4.4 ± 0.6
LVEF (%)	50 (15–80)
EUROScore II	1.98 (0.7–72)
Surgical procedure (*n*, %)	
AVR	54 (54)
AVR + CABG	32 (32)
AVR + MRV	1 (1)
AVR + MRV + CABG	2 (2)
TAVI	12 (12)
Comorbid diseases (%)	
Diabetes	30
Hypertension	93
Hypercholesterolemia	24
Chronic renal disease	13
Chronic obstructive pulmonary disease	9
Coronary artery disease	70
Chronic heart failure	10
Peripheral vascular diseases	3
History of myocardial infarction	3
History of stroke	2

AVA—aortic valve area, AVR—aortic valve replacement, CABG—coronary artery bypass graft, CCI—Charlson Comorbidity Index, LVEF—left ventricular ejection fraction, MVR—mitral valve replacement, TAVI—transcatheter aortic valve implantation.

**Table 2:** Nutritional status and biochemical parameters of patients with AS before surgery (*n* = 101).

Patients with AS (*n* = 101)	Mean ± SD or Median and Range
f-MNA	24.3 ± 2.55
7-SGA	6 (2-6)
SNAQ	15.76 ± 1.8
BMI (kg/m^2^)	28.9 ± 5.7
HGS (kg)	26.5 ± 9.6
Weight loss (%)	0 (0–11)
Phase angle (50 kHz)	8.7 ± 1.8
Biochemical parameters	
Albumin (g/L)	36.7 ± 6.7
Prealbumin (mg/dL)	31.83 ± 7.03
White blood cells (×109/L)	7.6 ± 2.1
Total number of lymphocytes (/mm^3^)	1.8 ± 0.6
Red blood cells (×109/L)	4.4 ± 0.5
Hemoglobin (mg/dL)	13.2 ± 1.4
CRP (mg/dL)	1.73 (0.01–18.4)
HDL-cholesterol (mg/dL)	47.9 ± 14.4
LDL-cholesterol (mg/dL)	67.5 (23–177)
Total cholesterol (mg/dL)	144.1 ± 42.1

BMI—body mass index, CRP—C-reactive protein, f-MNA—full Mini Nutritional Assessment, HDL—high-density cholesterol, HGS—handgrip strength, LDL—low-density lipoprotein, SGA—Subjective Global Assessment, SNAQ—Simplified Nutritional Appetite Questionnaire.

**Table 3:** Predictive value of nutritional status parameters for postoperative complications.

*n* = 101	AUC	SE	AUC Lower 95%	AUC Upper 95%	*p*-Value
f-MNA	0.624	0.056	0.514	0.734	0.027
7-SGA	0.374	0.053	0.269	0.479	0.023
SNAQ	0.428	0.055	0.320	0.537	0.195

AUC—area under the curve; f-MNA—full Mini Nutritional Assessment; SE—standard error; SGA—Subjective Global Assessment; SNAQ—Simplified Nutritional Appetite Questionnaire.

**Table 4:** Predictive value of biochemical parameters for postoperative complications.

*n* = 101	AUC	SE	AUC Lower 95%	AUC Upper 95%	*p*-Value
Total cholesterol (mg/dL)	0.642	0.078	0.49	0.795	0.007
LDL-cholesterol (mg/dL)	0.668	0.08	0.511	0.826	0.036
Prealbumin (mg/dL)	0.668	0.075	0.52	0.816	0.025

AUC—area under the curve; LDL—low-density lipoprotein; SE—standard error.

**Table 5:** The comparison between patients who developed postoperative complications (complicated) and patients without postsurgical complications (uncomplicated).

Data Presented as mean ± SD or Median and Range	Complicated (*n* = 50)	Uncomplicated (*n* = 51)	*p*-Value
Female/male (*n*)	22/28	26/25	0.434
Age (years)	75.5 ± 5.4	73.9 ± 5	0.062
Age adjusted CCI	5 (2–9)	4 (3–8)	0.308
Hospital length of stay (days)	11.1 ± 5.8	8.8 ± 3.8	0.038
LVEF (%)	50 ± 9	53.4 ± 8	0.079
AVA (cm^2^)	0.735 ± 0.18	0.889 ± 0.9	0.659
Flow speed (m/s)	4.4 ± 0.7	4.4 ± 0.5	0.907
Mean gradient (mm Hg)	47.5 ± 12.9	46.8 ± 13	0.928
EUROScore II	2.3 (0.8–7.2)	1.9 (0.7–6.2)	0.149
6-month weight loss (%)	1.9 (0–9)	1.5 (0–11.3)	0.459
BMI (kg/m^2^)	28.7 ± 5.9	28.9 ± 5.5	0.975
f-MNA	23.7 ± 2.7	25 ± 2.3	0.033
7-SGA	5 (2–6)	6 (3–6)	0.034
SNAQ	15.7 ± 2	15.9 ± 1.6	0.487
HGS (kg)	25.3 ± 9.0	27.9 ± 9.9	0.170
Phase angle (50kHZ)	8.5 ± 1.32	8.9 ± 2.1	0.696
Albumin (g/L)	35.7 ± 6.5	38.3 ± 7.4	0.230
Prealbumin (mg/dL)	29.73 ± 8.23	34.4 ± 7.7	0.038
CRP (mg/dL)	1.29 (0.01– 12.49)	2.3 (0.01–18.4)	0.364
LDL-cholesterol (mg/dL)	53.5 (23–177)	71 (43–112)	0.039
Total cholesterol (mg/dL)	134.03 ± 47.8	148.4 ± 35.8	0.076
Total number of lymphocytes (/mm^3^)	1.7 ± 0.6	1.9 ± 0.6	0.054
Haemoglobin (g/dL)	13.15 ± 1.54	13.3 ± 1.4	0.495

BMI—body mass index, CCI—Charlson Comorbidity Index, CRP—C-reactive protein, HGS—handgrip strength, LDL—low-density lipoprotein, LVEF—left ventricular ejection fraction, f-MNA—full Mini Nutritional Assessment, SGA—Subjective Global Assessment, SNAQ—Simplified Nutritional Appetite Questionnaire.
